# Adaptive State-Separated UFIR Filter for Attitude Estimation Using MARG Sensors

**DOI:** 10.3390/mi17020174

**Published:** 2026-01-28

**Authors:** Zepeng Li, Yuhang Zhu, Zheng Zhou, Shunyi Zhao

**Affiliations:** Key Laboratory of Advanced Process Control for Light Industry (Ministry of Education), Jiangnan University, Wuxi 214122, China; zepeng.li@stu.jiangnan.edu.cn (Z.L.); yuhang.zhu@stu.jiangnan.edu.cn (Y.Z.); z.zhou@stu.jiangnan.edu.cn (Z.Z.)

**Keywords:** magnetic, angular rate, and gravity (MARG), attitude estimation, unbiased finite impulse response (UFIR) filter, quaternions, adaptive

## Abstract

Unbiased Finite Impulse Response (UFIR) filters are widely used in engineering applications, such as vehicle attitude estimation, due to their advantages, including independence from initial conditions and insensitivity to noise. However, the performance of the UFIR filter heavily relies on the estimation horizon *N*, and different states within the system may exhibit an inverse correlation with respect to *N*, affecting the estimation results. To address this issue, this paper proposes an adaptive state-separated UFIR (ASSUFIR) filtering algorithm based on the properties of quaternions. By leveraging the relationship between quaternions and attitude angles, the algorithm reduces the computational burden of the batch UFIR filter estimation system, allowing different horizon lengths to be applied to different states. To mitigate the computational efficiency loss caused by disrupting the original UFIR filter structure, QR decomposition is introduced. The algorithm is first validated using simulated data and then compared with classical methods using real vehicle data. Experimental results demonstrate the practical applicability of the proposed method in engineering applications.

## 1. Introduction

In recent years, driven by rapid advances in autonomous driving and intelligent mobility, accurate ground-vehicle attitude estimation (pitch, roll, and yaw) has become critical for navigation, perception, stability control, and safety functions in challenging operating environments [1,2,3,4,5,6]. In parallel, continued progress in micro-electro-mechanical systems (MEMS) has enabled lightweight inertial sensors and multi-sensor integration to be widely deployed for attitude estimation due to their favorable dynamic response and ease of integration [7,8,9,10].

A magnetic, angular-rate, and gravity (MARG) sensing unit, typically composed of an inertial measurement unit (IMU) and a triaxial magnetometer, offers a compact, low-power solution. The IMU provides high-rate angular-rate and specific-force measurements, while the magnetometer supplies a heading reference through the local geomagnetic field. In practice, however, sensor imperfections and environmental disturbances (e.g., gyroscope bias drift, maneuver-induced specific-force acceleration, and magnetic distortions) can substantially degrade estimation accuracy [11,12,13]. As a result, robust attitude estimation generally requires sensor fusion frameworks that integrate multi-modal information.

Research on MARG-based attitude estimation can be broadly categorized into (i) attitude reconstruction methods and (ii) filtering/observer-based methods. Reconstruction approaches estimate attitude by solving the Wahba problem using vector observations derived from accelerometer and magnetometer measurements [14,15,16]. Filtering-based approaches incorporate process models and measurement updates to produce smoothed, dynamically consistent attitude estimates [17,18].

Among filtering approaches, Kalman-filter-based methods are widely used due to their strong estimation performance. For linear systems with independent white Gaussian noise, the Kalman filter is statistically optimal and often converges rapidly in practice. To accommodate quaternion representations and address nonlinearity, various quaternion-based formulations and Kalman filter variants have been developed, including the extended Kalman filter (EKF) [19] and indirect Kalman filter (IKF) [20]. Computationally efficient variants, such as lightweight EKF, have also been proposed to reduce the cost of the update step [21], while multi-layer or cascaded architectures can improve robustness under modeling mismatch.

Nevertheless, Kalman-filter-based methods typically depend on accurate process or measurement models and reliable noise statistics [22,23,24], which are difficult to obtain in highly dynamic, disturbance-prone conditions common in ground-vehicle operation. To reduce reliance on noise statistics, finite-impulse-response (FIR) approaches have been explored. In particular, the unbiased FIR (UFIR) filter estimates the current state from a finite window of recent measurements and does not require explicit noise statistics or precise initial conditions [25]. A key design parameter is the horizon length. For time-invariant systems, $N_{\mathrm{opt}}$ can be selected by minimizing the mean-square error (MSE) under test conditions [26,27]. For time-varying systems, however, a fixed horizon may fail to track changing dynamics, motivating adaptive horizon-selection strategies. Existing studies have proposed maximum-likelihood-based selection [28] and filter-bank approaches for real-time horizon adjustment [29].

**Figure 1:**
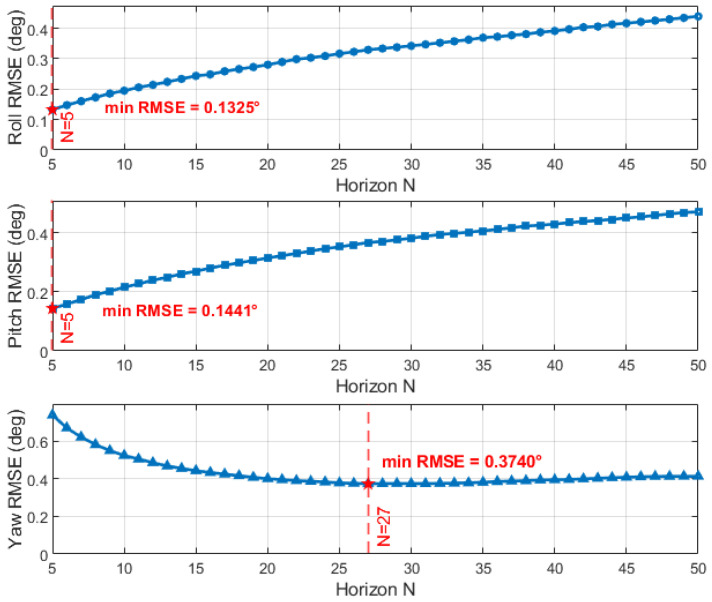
Different components exhibit distinct sensitivities to the UFIR horizon length *N*.

Building on our previous quaternion-based UFIR attitude estimation framework, we identify two practical issues in dynamic vehicle operation. First, because the state model is driven by gyroscope integration, the effective system behavior is time-varying; consequently, a single fixed horizon length *N* is only optimal at certain time instants. Second, empirical results indicate that the attitude components can exhibit conflicting sensitivity to *N*: for example, horizon lengths that improve estimation for one angle (e.g., pitch/roll) may degrade another (e.g., yaw), leading to a compromise when a single global horizon is used (see Figure 1).

Motivated by these observations, we propose a quaternion-based adaptive state-separated UFIR (ASS-UFIR) method. The main contributions are:

(1) We reformulate the extended state-space model by exploiting quaternion properties, enabling different horizon lengths to be assigned to different attitude-related state components while maintaining a consistent quaternion representation (e.g., through appropriate constraint handling/normalization). This mitigates conflicting horizon sensitivity across attitude angles.
(2) Empirically, the UFIR performance varies nonlinearly with *N* and often exhibits a local-optimal region in which small horizon changes have a limited effect. Based on this observation, we construct an offline admissible interval for *N* and map an estimated motion regime to this interval to update *N* online at each step, enabling adaptive selection with low tuning burden.
(3) Because state-dependent horizons disrupt the standard iterative UFIR structure, we adopt a batch least-squares formulation. To reduce computation and avoid explicit matrix inversion, we implement batch updates using a QR-decomposition-based solver, thereby improving numerical stability and runtime efficiency for embedded systems.

The remainder of this paper is organized as follows. Section 2 reviews related work and presents the system model and notation used throughout the paper. Section 3 describes the proposed adaptive state-separated UFIR filtering method, including its design rationale and implementation. Section 4 develops an efficient QR-decomposition-based solution for the resulting batch formulation. Section 5 presents simulation and real-vehicle experimental results, and compares the proposed method with representative baseline approaches. Finally, Section 6 concludes the paper and discusses directions for future work.

## 2. Preliminaries

To support quaternion-based three-dimensional attitude representation, this section summarizes the quaternion definitions, notation, and key properties used throughout the paper, and then presents the mathematical models adopted in this study.

### 2.1. Attitude Representation

The attitude of a rigid body describes the orientation of the body-fixed coordinate frame *b* with respect to the navigation frame *n*. The *b*-frame is rigidly attached to the vehicle (or mobile robot) and its axes are aligned with the vehicle structure, whereas the *n*-frame serves as a reference frame aligned with the chosen navigation convention (e.g., a local-level geographic frame), providing an absolute directional reference.

The relative rotation between any two frames can be represented by a quaternion. Let the quaternion be expressed as

(1) $$\begin{array}{c} q={\left[q_{0},\;q_{1},\;q_{2},\;q_{3}\right]}^{⊤}, \end{array}$$

where $q_{0}$ denotes the real part and ${\left[q_{1},q_{2},q_{3}\right]}^{⊤}$ denotes the imaginary part. According to the quaternion rotation definition, it can be written as

(2) $$\begin{array}{c} q=\left[\begin{array}{c} q_{0}\\ q_{1}\\ q_{2}\\ q_{3} \end{array}\right]=\left[\begin{array}{c} \cos\;\left(\alpha /2\right)\\ u_{x}\sin\;\left(\alpha /2\right)\\ u_{y}\sin\;\left(\alpha /2\right)\\ u_{z}\sin\;\left(\alpha /2\right) \end{array}\right], \end{array}$$

where α is the rotation angle, and $u={\left[u_{x}\;u_{y}\;u_{z}\right]}^{⊤}$ is the unit vector representing the rotation axis. Because quaternions encode rotations, unit normalization is commonly enforced to avoid representation ambiguity and to simplify subsequent computation. Moreover, the conjugate corresponds to the opposite rotation as

(3) q*nb=qbn=[q0,−q1,−q2,−q3]⊤.

### 2.2. Problem Formulation

To facilitate the discussion of the proposed method, it is essential to define the notations and coordinate systems used throughout this paper. The sensor coordinate system is designated as the body frame (*b*), and the global reference coordinate system is denoted as the navigation frame (*n*). The measurement of the accelerometer is defined as ab=[axayaz]⊤, and the gravitational acceleration is given by gn=[001]⊤. In these expressions, $a_{x}$, $a_{y}$, and $a_{z}$ are the components of acceleration along the three axes of the body frame.

Measurements from MARG sensors are typically non-normalized vectors with differing units (e.g., meters per second squared for acceleration and Gauss for magnetic field strength). These inconsistencies in scale can pose challenges during data fusion. Similarly, the magnetometer measurement is defined as mb=[mxmymz]⊤ with ||mb||=1. The reference vector for the horizontal component of the magnetic field is given by hn=[hxhyhz]⊤ with hn=1. Finally, the measurement of the gyroscope is defined as ωb=[ωxωyωz]⊤.

### 2.3. Construction of the State-Space Model

In this study, we adopt the algebraic quaternion model proposed in [18] to formulate the system dynamics and measurements. The system state is denoted by the quaternion q, where *k* and k−1 represent the current and previous time steps, respectively. Based on this model, the state and measurement equations are

(4) $$\begin{array}{c} q_{k}=\Phi_{k}q_{k-1}+\eta_{k}, \end{array}$$

(5) $$\begin{array}{c} q_{k}^{\mathrm{meas}}=H_{k}q_{k}+\xi_{k}. \end{array}$$

Here, $\Phi_{k}$ represents the state transition matrix, obtained from the quaternion differential equation via a first-order discretization [30]

(6) $$\Phi_{k}=\frac{\Delta t}{2}\left[\Omega \times \right]+I,$$

where Δt denotes the sampling interval. The operator [Ω×] is the quaternion-rate matrix constructed from ωbk as

(7) $$\left[\Omega \times \right]=\left[\begin{array}{cccc} 0 & -\omega_{x,k} & -\omega_{y,k} & -\omega_{z,k}\\ \omega_{x,k} & 0 & \omega_{z,k} & -\omega_{y,k}\\ \omega_{y,k} & -\omega_{z,k} & 0 & \omega_{x,k}\\ \omega_{z,k} & \omega_{y,k} & -\omega_{x,k} & 0 \end{array}\right].$$

And $H_{k}$ is the observation matrix and is set to the identity matrix. The vector $q_{k}^{\mathrm{meas}}$ denotes the measurement, and $q_{k}^{\mathrm{meas}}$ is the algebraic quaternion derived analytically from the accelerometer and magnetometer [18]. The specific derivation process is not the focus of this article, and the conclusion is estimated to be directly given here:

(8) $$q_{k}^{\mathrm{meas}}=q_{\mathrm{acc}}⊗q_{\mathrm{mag}}.$$

Among them, ⊗ denotes quaternion multiplication, and $q_{\mathrm{acc}}$ and $q_{\mathrm{mag}}$ are quaternions obtained through algebraic analysis of measurement data from accelerometers and magnetometers, respectively. The terms $\eta_{k}$ and $\xi_{k}$ are the process and measurement noise, respectively.

## 3. Adaptive State-Separated UFIR Filter

### 3.1. State-Separated UFIR Filter Design

**Figure 2:**
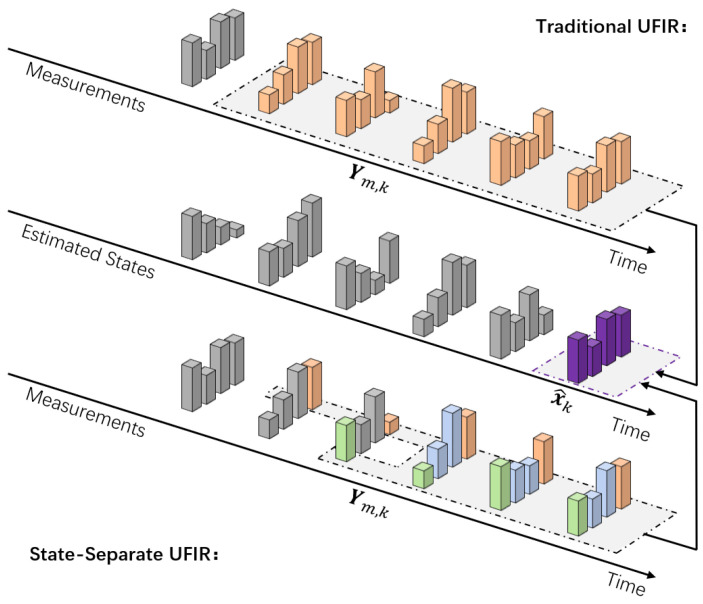
Structural illustration of the proposed SS-UIFR and traditional UFIR. The measurement values in the first row represent the structure of the estimation results obtained by traditional UFIR, while the third row represents the structure of the estimation results obtained by the proposed SS-UFIR.

In our previous work, we reformulated a quaternion-based linear attitude estimation model from the KF framework into the UFIR framework and experimentally validated improved robustness. However, we observed that the horizon length can affect different state components in different (and sometimes opposing) ways. Consequently, using a single global horizon for all components may yield only locally optimal performance for certain attitude components while degrading others. To address this issue, we propose a state-separated UFIR (SS-UFIR) method based on an extended state-space formulation. By partitioning the estimation problem into reduced sub-models associated with different state components, the proposed approach allows each component (or component group) to employ a distinct horizon length *N*. This design aims to improve overall attitude estimation accuracy across all components. The structural difference between the conventional global-horizon strategy and the proposed state-separated strategy is illustrated in Figure 2. Consider the discrete-time linear model (without control input) introduced above. For a given time index *k*, define the horizon [m,k], with m=k−N+1, so that the horizon contains *N* consecutive samples. The specific value of *N* can be referred to [25]. The corresponding extended state-space model over [m,k] can then be written as follows:

(9) $$\begin{array}{cc} X_{m,k} & =F_{m,k}q_{m}+W_{m,k}, \end{array}$$

(10) $$\begin{array}{cc} Y_{m,k} & ={\bar{H}}_{m,k}q_{m}+{\bar{C}}_{m,k}W_{m,k}+V_{m,k}. \end{array}$$

The components of the extended state-space system are defined as follows:

(11) $$\begin{array}{cc} X_{m,k} & ={\left[\begin{array}{cccc} q_{m}^{⊤} & q_{m+1}^{⊤} & \ldots & q_{k}^{⊤} \end{array}\right]}^{⊤}, \end{array}$$

(12) $$\begin{array}{cc} Y_{m,k} & ={\left[\begin{array}{cccc} {\left(q_{m}^{\mathrm{meas}}\right)}^{⊤} & {\left(q_{m+1}^{\mathrm{meas}}\right)}^{⊤} & \ldots & {\left(q_{k}^{\mathrm{meas}}\right)}^{⊤} \end{array}\right]}^{⊤}, \end{array}$$

(13) $$\begin{array}{cc} W_{m,k} & ={\left[\begin{array}{cccc} \eta_{m}^{⊤} & \eta_{m+1}^{⊤} & \ldots & \eta_{k}^{⊤} \end{array}\right]}^{⊤}, \end{array}$$

(14) $$\begin{array}{cc} V_{m,k} & ={\left[\begin{array}{cccc} \xi_{m}^{⊤} & \xi_{m+1}^{⊤} & \ldots & \xi_{k}^{⊤} \end{array}\right]}^{⊤}, \end{array}$$

(15) $$\begin{array}{cc} F_{m,k} & ={\left[\begin{array}{ccccc} I & \Phi_{m+1}^{⊤} & \ldots & {\left(\phi_{k-1}^{m+1}\right)}^{⊤} & {\left(\phi_{k}^{m+1}\right)}^{⊤} \end{array}\right]}^{⊤}, \end{array}$$

(16) $$\begin{array}{cc} {\bar{C}}_{m,k} & =\mathrm{diag}\left(\begin{array}{cccc} H_{m} & H_{m+1} & \ldots & H_{k} \end{array}\right)=I, \end{array}$$

(17) $$\begin{array}{cc} \phi_{i}^{j} & =\left\{\begin{array}{cc} \Phi_{i}\Phi_{i-1}\ldots \Phi_{j}, & j<i+1,\\ \;\;I, & j=i+1,\\ \;\;0, & j>i+1, \end{array}\right. \end{array}$$

in which ${\bar{H}}_{m,k}={\bar{C}}_{m,k}F_{m,k}$. Subsequently, according to the unbiasedness condition and zero-input response, we define the $H_{m,k}={\bar{H}}_{m,k}{\left(\phi_{k}^{m+1}\right)}^{-1}$. Due to the system defined in this study having no input, the generalized noise power gain (GNPG) [25] is given by

(18) $$\begin{array}{cc} G_{k} & ={\left(H_{m,k}^{⊤}H_{m,k}\right)}^{-1}. \end{array}$$

The batch UFIR filter then calculates the state estimates at time epoch *k* as follows:

(19) $${\hat{q}}_{k}=G_{k}H_{m,k}^{⊤}Y_{m,k}.$$

By substituting $G_{k}$ into the equation and rearranging the terms, the expression can be written in the standard normal equation form as follows:

(20) $$H_{m,k}\;{\hat{q}}_{k}=Y_{m,k}.$$

According to the characteristics of our model, since the system has no input and the measurement matrix is an identity matrix, expanding the above equation yields a homogeneous linear overdetermined system with a size of *N* × 4 (where 4 is the state dimension), as shown below:

(21) $$\begin{array}{c} \underset{H_{m,k}\in R^{4N\times 4}}{\underset{︸}{\left[\begin{array}{c} C_{m}{\left(\phi_{k}^{m+1}\right)}^{-1}\\ C_{m+1}{\left(\phi_{k}^{m+2}\right)}^{-1}\\ \vdots \\ C_{k-1}\Phi_{k}^{-1}\\ C_{k} \end{array}\right]}}\;\;{\hat{q}}_{k}=\underset{Y_{m,k}\in R^{4N\times 1}}{\underset{︸}{\left[\begin{array}{c} q_{m}^{\mathrm{meas}}\\ q_{m+1}^{\mathrm{meas}}\\ \vdots \\ q_{k-1}^{\mathrm{meas}}\\ q_{k}^{\mathrm{meas}} \end{array}\right]}}. \end{array}$$

We now return to the definition of quaternions. According to their mathematical formulation, the four elements of a quaternion respectively represent the rotation angle and the orientation of the rotation axis along three spatial directions. In the defined local coordinate frame, the last three components can be interpreted as the rotational components corresponding to the body’s three principal axes. Hence, these imaginary components can be generally regarded as carrying the primary rotational information of the rigid body’s attitude. Based on this understanding, the final attitude angles estimated by the filter are mapped onto the system state, enabling a generalized separation among the three attitude angles.

When incorporating this idea into the UFIR-based attitude estimation process, we introduce two horizon lengths, $N_{p}$ and $N_{y}$, to reflect different filtering requirements. Let $N_{\max}=\max\left\{N_{p},N_{y}\right\}$ and $N_{\min}=\min\left\{N_{p},N_{y}\right\}$. The larger horizon $N_{\max}$ is used for state components that benefit from stronger noise suppression and smoother estimates, whereas the smaller horizon $N_{\min}$ is used for components requiring faster dynamic response.

In the model considered in this study, as shown in Equations (6) and (7), the measurements from the gyroscope form the state transition equation and have already been integrated into the system’s prior calculation as part of the state prediction. On the other hand, as shown in Equation (8), the observation quaternion is constructed from the measurements of the accelerometer and magnetometer, and they are fused through quaternion multiplication. Before fusion, the decomposed quaternion definitions are as follows: $q_{\mathrm{mag}}$ represents the rotation around the *z*-axis, meaning that the magnetometer is primarily used to provide heading reference. The accelerometer, on the other hand, is used to provide pitch and roll information. Therefore, in subsequent analysis, we group pitch and roll together, assigning them the horizon $N_{p}$. Yaw is treated separately, using the horizon $N_{y}$.

Under the adopted small-angle (or linearized) quaternion model, these groupings correspond to assigning $N_{p}$ to the roll/pitch-related quaternion components (e.g., $q_{1}$ and $q_{2}$) and to the yaw-related component (e.g., $q_{3}$). During the batch UFIR solution at time step, the stacked equation system can be truncated according to the horizon associated with each state group, i.e., using measurements over $\left[k-N_{p}+1,k\right]$ for the roll/pitch group and $\left[k-N_{y}+1,k\right]$ for the yaw group. To reduce the start-up delay, we initialize the horizon as $N_{\mathrm{init}}=N_{\min}$, enabling an earlier valid estimate and shortening the initial warm-up period. Index stacked rows in time major order by

(22) $$\begin{array}{c} r(t,c)=4\;(t-m)+c,\; \end{array}$$

where t∈{m,…,k},c∈{1,2,3,4}, and the time set retained by each dimension is

(23) $$\begin{array}{cc} D^{\left(1\right)} & =\underset{N}{\underset{︸}{\left\{\;m,\ldots ,k\;\right\}}},\;D^{\left(4\right)}=\underset{N_{y}}{\underset{︸}{\left\{\;k-N_{y}+1,\ldots ,k\;\right\}}},\\ D^{\left(2\right)} & =D^{\left(3\right)}=\underset{N_{p}}{\underset{︸}{\left\{\;k-N_{p}+1,\ldots ,k\;\right\}}}, \end{array}$$

where $D^{\left(c\right)}$ denotes the set of horizons in the *c*-th dimension of a quaternion, $N=N_{\max}$. Mapping these time sets into a set of row numbers and merging them together is the preserved set of rows:

(24) $$\begin{array}{c} R_{\mathrm{keep}}\;=\;\overset{4}{\underset{c=1}{⋃}}\;\left\{\;r\left(t,c\right)\;:\;t\in D^{\left(c\right)}\;\right\}. \end{array}$$

The number of reserved rows (i.e., the number of equations after clipping):

(25) $$\begin{array}{cc} M\;=\;|R_{\mathrm{keep}}|\; & =\;4N-2\left(N-N_{p}\right)-\left(N-N_{y}\right)\\ \; & =\;N\;+\;2N_{p}\;+\;N_{y}. \end{array}$$

Subsequently, construct the trimming (row-selection) matrix $S_{r}$ by stacking standard-basis row vectors. Let ${\left\{e_{i}\right\}}_{i=1}^{4N}$ be the standard basis of $R^{4N}$, where $e_{i}$ is the column vector with a single 1 in the *i*-th position and zeros elsewhere. Sort the elements of $R_{\mathrm{keep}}$ in ascending order as $R_{\mathrm{keep}}={\left\{i_{j}\right\}}_{j=1}^{M}$, where $i_{j}$ represents the *j*-th element of the set, and *M* is the total number of reserved rows. Then, $S_{r}$ is constructed as follows:

(26) $$\begin{array}{c} S_{r}\;=\;{\left[\begin{array}{cccc} e_{i_{1}}^{⊤} & e_{i_{2}}^{⊤} & \ldots & e_{i_{M}}^{⊤} \end{array}\right]}^{⊤}\in {\left\{0,1\right\}}^{M\times 4N}. \end{array}$$

By construction, left-multiplication simply picks those rows:

(27) $$\begin{array}{cc} {\tilde{H}}_{m,k} & =S_{r}\;H_{m,k}\in R^{M\times 4}, \end{array}$$

(28) $$\begin{array}{cc} {\tilde{Y}}_{m,k} & =S_{r}\;Y_{m,k}\in R^{M\times 4}. \end{array}$$

Then, obtain the estimation result by solving the following equation:

(29) $${\tilde{H}}_{m,k}\;{\hat{q}}_{k}={\tilde{Y}}_{m,k}.$$

**Figure 3:**
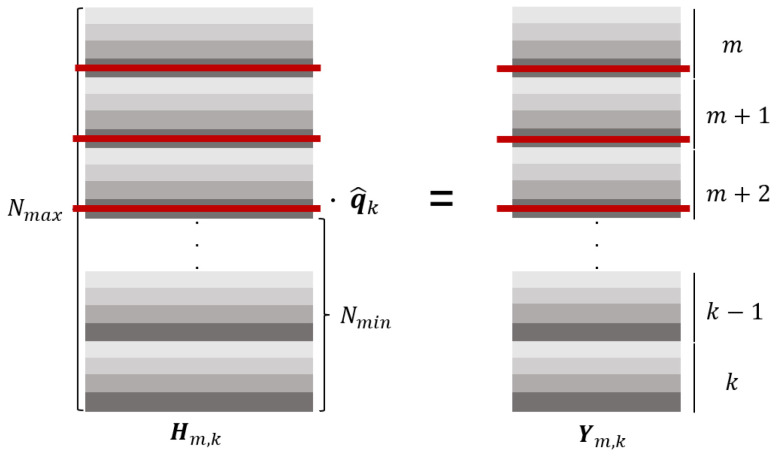
The pruning method for overdetermined equations. The different depths of gray here represent the four single states of quaternions, where red represents the size being trimmed (taking the yaw dimension as an example).

The diagram of the trimming process is shown in the Figure 3. Four different colors represent the quaternion components at different time steps, and the red lines indicate the trimmed sections. The figure uses yaw as an example with a small window.

### 3.2. Adaptive Horizon Length

As mentioned in [25], the only tuning parameter in the UFIR filter algorithm is the horizon length *N*, and its optimal selection is critical for minimizing the system’s MSE. Some studies suggest that, when test measurements are available, *N* can be optimized by minimizing the trace of the system’s error covariance matrix. Another approach, as discussed in [26], is to estimate *N* using the measurement values by minimizing the residual covariance matrix. However, these methods are primarily applicable to time-invariant systems. For time-varying systems, *N* must be dynamically adjusted at each time index *k*.

The constructed system, as shown in the (4) and (5), features a state transition matrix derived from the gyroscope’s differential equation. The core structure is based on the gyroscope measurements, classifies it as time-varying. Therefore, to maintain the estimation performance of the system, the value of *N* must be dynamically updated at each time step.

In the past, several methods for adaptively selecting have been proposed, mainly based on maximum likelihood estimation or the UFIR bank approach. The essence of these methods is to set an evaluation metric at each time step and compare the system’s estimation results under different values of *N*, selecting the optimal outcome. For some embedded devices, these methods are time-consuming and require substantial memory, which may not be feasible due to memory constraints. To address this, this paper proposes a novel method for adaptive adjustment of *N* based on a linear mapping range.

As shown in the Figure 1, the relationship between the system’s estimation performance and *N* is nonlinear, and the effect of local variations of *N* on the system performance can be almost negligible. Based on this characteristic, we determine an acceptable range for *N* offline, namely $N\in [N_{\min},N_{\max}]$, where $N_{\min}$ and $N_{\max}$ are the minimum and maximum values derived from offline testing.

Next, based on the system motion characteristics, we define two angular–rate thresholds to the different state components, $\theta_{\mathrm{pitch}}$ and $\theta_{\mathrm{yaw}}$, where $\theta_{\mathrm{pitch}}$ denotes the pitch and roll threshold, and $\theta_{\mathrm{yaw}}$ characterizes yaw variations. Instead of relying on a single-step difference, we first compute the instantaneous angular rates at each sample and then average them over the window of length *N*:

(30) $$\begin{array}{cc} {\bar{\dot{\theta }}}_{\mathrm{pitch}}\left(k\right) & =\frac{1}{N}\sum_{i=k-N+1}^{k}\frac{|\theta_{\mathrm{pitch}}\left(i\right)-\theta_{\mathrm{pitch}}\left(i-1\right)|}{\Delta t}, \end{array}$$

(31) $$\begin{array}{cc} {\bar{\dot{\theta }}}_{\mathrm{yaw}}\left(k\right) & =\frac{1}{N}\sum_{i=k-N+1}^{k}\frac{|\theta_{\mathrm{yaw}}\left(i\right)-\theta_{\mathrm{yaw}}\left(i-1\right)|}{\Delta t}. \end{array}$$

These averaged rates are then mapped to the UFIR horizons:

(32) $$\begin{array}{cc} N\ldots \left(k\right) & =N_{\min}+\left(N_{\max}-N_{\min}\right)\;\;\left(\frac{\left|\bar{\dot{\theta }}\ldots \left(k\right)\right|\Delta t}{\theta \ldots }\right), \end{array}$$

where {…} denotes the pitch or yaw. At the same time, we also need to normalize the mapping of proportions to between [0,1] in our calculations. Subsequently, we obtained two different horizons for trimming the number of equations in the UFIR filter. This update scheme can ensure the required dynamism of the window for time-varying systems, which helps to adaptively adjust the window size of the UFIR filter and optimize its performance.

## 4. QR Decomposition

As described in Section 3, introducing state-dependent horizon lengths enables state separation by reducing and restructuring the original UFIR estimation system. However, using multiple horizons breaks the shift-invariant structure exploited by the standard low-complexity iterative UFIR recursion. In particular, for the state group associated with the larger horizon $N_{\max}$, the corresponding stacked system contains an initial block of rows for which the required past information is not available (i.e., the recursion cannot be initialized in the same manner as in previous iterative UFIR formulations). Therefore, we adopt the batch UFIR formulation for attitude estimation. The batch formulation requires solving a large-scale overdetermined linear system at each time step (or over each sliding horizon). A direct solution based on explicit matrix inversion, e.g., forming and inverting the normal matrix, incurs substantial computational cost and may suffer from numerical ill-conditioning, especially on platforms with limited compute resources. Consequently, an efficient solver that avoids explicit inversion while maintaining estimation accuracy is needed. This motivates the use of QR decomposition in this study [31].

Following Equation (29), after stacking the windowed equations and applying the state-separation (row-trimming) operation, the reduced overdetermined system can be written as follows:

(33) $${\tilde{Y}}_{m,k}={\tilde{H}}_{m,k}q_{k}.$$

Since M≫4 in general, Equation (33) is solved in the least-squares sense, yielding the batch UFIR estimate

(34) $$\begin{array}{cc} {\hat{q}}_{k} & =\arg\min_{q}\;{∥{\tilde{H}}_{m,k}q-{\tilde{Y}}_{m,k}∥}_{2}^{2} \end{array}$$

(35) $$\begin{array}{cc} \; & ={\left({\tilde{H}}_{m,k}^{⊤}{\tilde{H}}_{m,k}\right)}^{-1}{\tilde{H}}_{m,k}^{⊤}{\tilde{Y}}_{m,k}, \end{array}$$

which is consistent with Equation (29). Although (35) provides a closed-form solution, explicitly forming and inverting ${\tilde{H}}_{m,k}^{⊤}{\tilde{H}}_{m,k}$ is computationally demanding and may degrade numerical robustness when the stacked system becomes ill-conditioned. To avoid explicit inversion, we perform QR factorization on the specific matrix in Equation (29), i.e., ${\tilde{H}}_{m,k}$:

(36) $${\tilde{H}}_{m,k}=Q_{m,k}R_{m,k},$$

where $Q_{m,k}\in R^{M\times M}$ is orthogonal ($Q_{m,k}^{⊤}Q_{m,k}=I$) and $R_{m,k}\in R^{M\times 4}$ is upper trapezoidal. Substituting (36) into (34) and using the invariance of the *ℓ*_2_-norm under orthogonal transforms, we obtain the following:

(37) $$\begin{array}{cc} {∥{\tilde{H}}_{m,k}q-{\tilde{Y}}_{m,k}∥}_{2} & ={∥Q_{m,k}R_{m,k}q-{\tilde{Y}}_{m,k}∥}_{2}\\ \; & ={∥Q_{m,k}^{⊤}\;\left(Q_{m,k}R_{m,k}q-{\tilde{Y}}_{m,k}\right)∥}_{2}\\ \; & ={∥R_{m,k}q-Q_{m,k}^{⊤}{\tilde{Y}}_{m,k}∥}_{2}. \end{array}$$

Let $z_{m,k}=Q_{m,k}^{⊤}{\tilde{Y}}_{m,k}\in R^{M\times 1}$. Since ${\tilde{H}}_{m,k}$ has only 4 columns, the QR factorization yields $R_{m,k}\in R^{M\times 4}$ with an upper-trapezoidal structure: the first 4 rows form an effective 4×4 upper-triangular block, denoted by ${\bar{R}}_{m,k}\in R^{4\times 4}$, whereas the remaining (M−4) rows are reduced to (approximately) zero during the elimination. Accordingly, $z_{m,k}$ is partitioned conformably as $z_{m,k}={\left[{\bar{z}}_{m,k}^{⊤}\;\;z_{m,k}^{⊥⊤}\right]}^{⊤}$, where ${\bar{z}}_{m,k}\in R^{4\times 1}$ consists of the first four elements of $z_{m,k}$ and is used together with ${\bar{R}}_{m,k}$ to form a back-substitutable triangular system, while $z_{m,k}^{⊥}\in R^{(M-4)\times 1}$ collects the remaining elements and represents the component lying in the orthogonal complement of the column space of ${\tilde{H}}_{m,k}$ after the orthogonal transform (i.e., the part associated with the least-squares residual). Therefore, $R_{m,k}$ can be written as

(38) $$R_{m,k}=\left[\begin{array}{c} {\bar{R}}_{m,k}\\ 0 \end{array}\right],\;{\bar{R}}_{m,k}\in R^{4\times 4},$$

and the least-squares problem is equivalently reduced to solving the following upper-triangular linear system:

(39) $${\bar{R}}_{m,k}{\hat{q}}_{k}={\bar{z}}_{m,k},$$

from which the quaternion estimate ${\hat{q}}_{k}$ is obtained by back substitution.

## 5. Validations

### 5.1. Numerical Simulation

Before conducting experiments, we first validate the proposed algorithm through numerical simulations and compare it with the traditional UFIR filter algorithm. The true attitude $q_{\mathrm{true}}$ is generated by adding random noise to the Equation (4). Then, simulated accelerometer and magnetometer measurements were derived based on the true geomagnetic field and gravitational acceleration values provided by, respectively,

(40) ab=R(qture)gn+δacc,

(41) mb=R(qture)hn+δmag,

where $R(q_{\mathrm{ture}})$ is the true attitude obtained from the simulation, δab and δmb represent the measurement noise from the accelerometer and magnetometer, respectively. The noise characteristics are modeled as follows: Gaussian white distribution. In subsequent simulations, the noise density is set $\delta_{\mathrm{gyr}}=0.0035\;\mathrm{rad}/s$, $\delta_{\mathrm{acc}}=0.018\;{m/s}^{2}$, and $\delta_{\mathrm{mag}}=0.022\;\mathrm{Gauss}$. These values were determined based on empirical observations of the noise characteristics in the MARG sensor readings used in subsequent experiments. Specifically, the sensor was kept stationary, and multiple static measurements were collected, each lasting 10 min. The data from each collection was combined with the calibration noise values provided by the sensor’s factory specifications, and the average values were calculated. Then, the system state is converted into the attitude angle form through the following transformation:

(42) $$\begin{array}{c} \theta =\arctan\left(\frac{2(q_{0}q_{1}+q_{2}q_{3})}{1-2(q_{1}^{2}+q_{2}^{2})}\right), \end{array}$$

(43) $$\begin{array}{c} \phi =\arctan\left(\frac{2(q_{0}q_{2}-q_{3}q_{1})}{1-2(q_{2}^{2}+q_{3}^{2})}\right), \end{array}$$

(44) $$\begin{array}{c} \psi =\arctan\left(\frac{2(q_{0}q_{3}+q_{1}q_{2})}{1-2(q_{3}^{2}+q_{1}^{2})}\right), \end{array}$$

where θ,ϕ,ψ respectively represent roll, pitch, and yaw.

**Figure 4:**
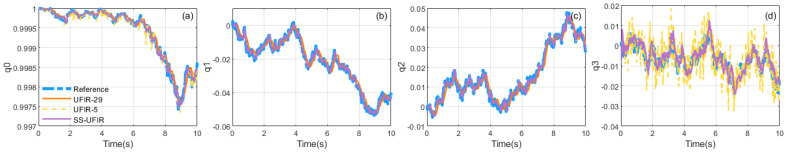
Comparison of quaternion-tracking accuracy under the same scale rotation: (**a**) $q_{0}$, (**b**) $q_{1}$, (**c**) $q_{2}$, (**d**) $q_{3}$, comparing the proposed UFIR-based estimator with the traditional UFIR.

**Figure 5:**
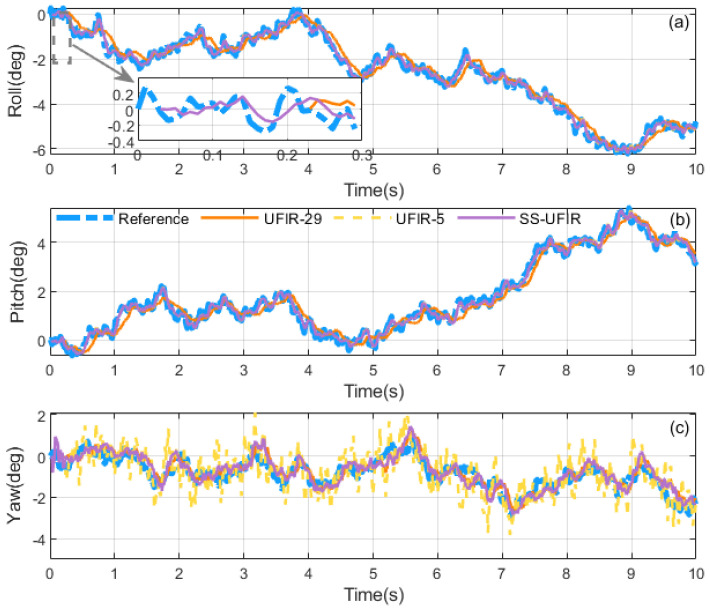
Comparison of attitude-tracking accuracy under the same scale rotation: (**a**) roll, where the thumbnail shows that the proposed algorithm has smaller gaps during the initialization phase, (**b**) pitch, (**c**) yaw, comparing the proposed estimator with the traditional UFIR.

First, we simulated a rotation with the same magnitude of motion, where the angular velocities along the *x*-, *y*-, and *z*-axes were randomly defined within the range 0.01×[−9,9] rad/s. The sampling frequency was set to 100 Hz, and the initial quaternion state was set as $q_{0}={\left[1,0,0,0\right]}^{⊤}$, with a 10-s simulation performed. During the testing phase, a representative set of simulated data was selected, and different horizon window sizes *N* were evaluated. For each candidate *N*, the corresponding UFIR estimation was performed, and the RMSE values of roll/pitch and yaw were computed, respectively. The optimal horizon for roll and pitch was chosen as the one that yielded the minimum RMSE, resulting in $N_{p}=5$, while the optimal horizon for yaw was selected in the same manner, giving $N_{y}=29$. These optimal horizon settings were then adopted in the subsequent algorithm comparisons. Therefore, traditional batch UFIR filters with fixed N=5 and N=29 were compared as benchmarks, and the results are shown in Figure 4 and Figure 5.

First, this simulation is designed not only for a statistical comparison of the final estimation results, but more importantly, to explicitly demonstrate how characteristic behaviors observed in the quaternion states are propagated to the attitude outputs through the quaternion-to-Euler mapping. Therefore, we present both the estimated state quaternions and the corresponding Euler-angle curves only in Figure 4 and Figure 5. Specifically, when a larger fixed horizon is adopted (e.g., UFIR-29), the estimation curves of $q_{1}$ and $q_{2}$ exhibit a pronounced lag, and the same lag is consistently reflected in the roll and pitch estimates; this lag is also observed in the subsequent simulations and experiments. In contrast, when a shorter horizon is used (e.g., UFIR-5), the $q_{3}$ component shows evident oscillations, which are mapped to the yaw channel and thus result in oscillatory yaw estimates. These results further verify the feasibility of the quaternion-to-Euler mapping for interpreting estimation behaviors. The above observations suggest that the three Euler-angle channels exhibit different sensitivities to the choice of the horizon length *N*, i.e., their estimation behaviors and error trends do not vary with *N* in a uniform manner. Consequently, adopting a single fixed-*N* UFIR filter may lead to an imbalanced performance across roll, pitch, and yaw, depending on the motion profile and measurement conditions. By contrast, the proposed SS-UFIR alleviates this intrinsic trade-off by assigning different horizon lengths to different state groups, thereby achieving a more balanced estimation behavior across all three axes.

To further quantify the overall attitude accuracy beyond the channel-wise Euler-angle RMSEs, we additionally introduce a global metric, namely the rotation angle (RA) error. Let $q_{\mathrm{true},k}$ and ${\hat{q}}_{k}$ denote the reference and estimated unit quaternions at time step *k*, respectively, and define the relative quaternion as $q_{e,k}=q_{\mathrm{true},k}^{-1}\;⊗\;{\hat{q}}_{k}$, where ⊗ denotes quaternion multiplication. The rotation angle error is then defined as

(45) $$\beta_{k}=2\arccos\;\left(\left|q_{e,k,0}\right|\right),$$

where $q_{e,k,0}$ is the scalar part of $q_{e,k}$ (the absolute value removes the sign ambiguity of unit quaternions). We report the RMSE of $\left\{\beta_{k}\right\}$ as a global performance indicator.

This conclusion is consistent with the quantitative results in Table 1: SS-UFIR achieves low roll/pitch errors comparable to UFIR-5 while maintaining a yaw error comparable to UFIR-29, thereby demonstrating superior overall performance.

Subsequently, the angular velocities along the *x*- and *y*-axes are defined as random values within the range 0.01×[−9,9] rad/s, while the angular velocity along the *z*-axis is defined as random values within the range 1×[−9,9] rad/s. This setup is designed to better simulate the actual motion scenarios of vehicles or mobile robots during the attitude estimation process. In this simulation, we introduced the method of adaptive adjustment of *N* based on the angular velocities and compared it with the traditional algorithm. The optimal values for the small and large windows were found to be 5 and 35, respectively.

**Figure 6:**
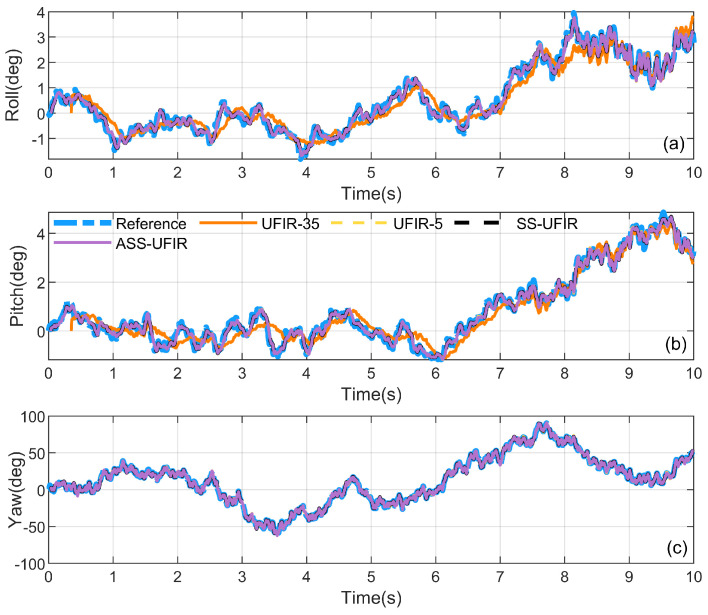
Comparison of attitude-tracking accuracy under simulated vehicle data: (**a**) roll, (**b**) pitch, (**c**) yaw, comparing the proposed estimator with the traditional UFIR.

As shown in Figure 6, the simulated vehicle-dynamics results indicate that the estimation behaviors of different attitude channels respond differently to the choice of horizon length *N*. Table 2 summarizes the Euler-angle RMSEs together with a global metric. For the fixed-horizon baselines, UFIR-5 achieves low roll/pitch errors (0.201°/0.190°) but yields relatively larger yaw (3.187°). Increasing the horizon (UFIR-37) slightly reduces the yaw error to 3.165°, yet it introduces a clear degradation in roll and pitch (0.330°/0.333°) and does not improve the global RA. By contrast, the proposed SS-UFIR provides a more favorable overall trade-off by assigning different horizon lengths to different state groups. As reported in Table 2, SS-UFIR preserves the low roll/pitch errors comparable to UFIR-5 (0.201°/0.190°), while reducing the yaw RMSE to 3.152° and the global RA to 3.161°, thereby indicating an overall improvement at the attitude level. Moreover, after incorporating the adaptive horizon mechanism, the same accuracy is achieved, which supports the effectiveness of the adaptive horizon selection in this setting.

Finally, Table 2 also reports the computation time, highlighting the advantage of the QR-based solver. Although ASS-UFIR/SS-UFIR require solving an overdetermined system at each time step, explicitly forming and inverting the normal matrix is unnecessary. By employing QR decomposition implemented via Givens rotations, ASSQR-UFIR reduces the computation time from 0.56s to 0.42s, corresponding to an improvement of approximately 25%, while maintaining identical RMSEs and RA to ASS-UFIR.

### 5.2. Field Experiment

**Figure 7:**
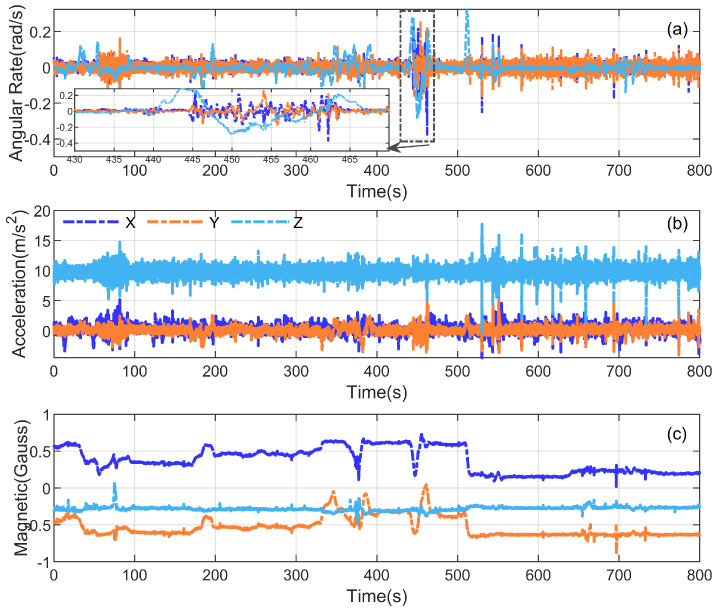
Raw measurement data from the three sensors: (**a**) three-axis outputs of the gyroscope, (**b**) three-axis outputs of the accelerometer, and (**c**) three-axis outputs of the magnetometer.

We evaluated the proposed adaptive state-separated QR-UFIR (ASSQR-UFIR) on approximately 800 s of real vehicle data; the raw IMU and magnetometer measurements are shown in Figure 7. Attitude estimation accuracy was assessed against a reference attitude obtained from an attitude and heading reference system (AHRS), and the attitude error was computed as Euler-angle differences. For benchmarking, we compared against the Madgwick gradient-descent attitude (GDA) filter, Mahony’s SO(3) complementary filter, AQUA-KF, and the conventional batch UFIR filter. For AQUA-KF, the initial quaternion and covariance were set to $q_{0}={\left[1,0,0,0\right]}^{⊤}$ and $\Sigma_{q_{0}}=I$, and the process/measurement noise parameters were set according to the sensor datasheet. The Madgwick gain was set to 0.08 following [32], and the Mahony proportional gain was set to 0.5 following the implementation in [33]. For the batch UFIR baseline, we report results for fixed horizons N=5 and N=70. For ASSQR-UFIR, we used $N_{\min}=5$ and $N_{\max}=70$, with $N_{\mathrm{init}}=5$ and $N_{\min}$.

**Figure 8:**
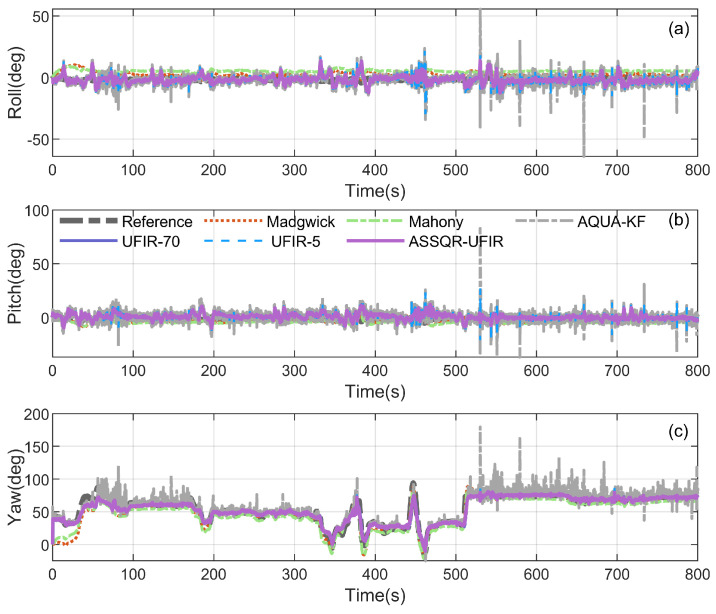
Attitude estimation results compared with classical methods: (**a**) roll, (**b**) pitch, (**c**) yaw.

**Figure 9:**
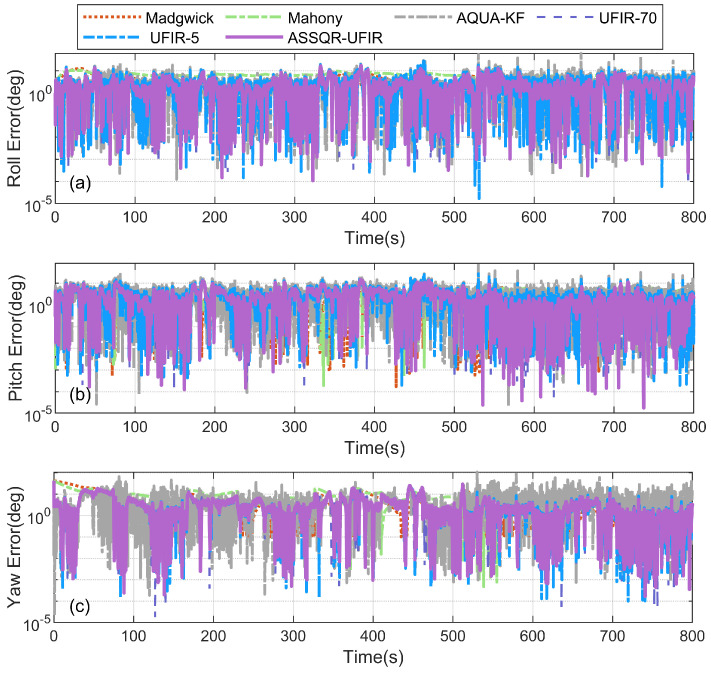
Attitude estimation errors in terms of Euler angles: (**a**) roll, (**b**) pitch, (**c**) yaw.

The estimated attitudes and the corresponding errors are illustrated in Figure 8 and Figure 9, and Table 3 reports the RMSEs, the proposed global metric, and the runtime. Overall, ASS-UFIR provides the most favorable accuracy profile in this experiment: it achieves consistently low RMSEs in roll, pitch, and yaw, and simultaneously yields the smallest RA RMSE (6.923°), indicating superior overall attitude consistency on SO(3) rather than only channel-wise improvements. Compared with fixed-horizon UFIR baselines, the state-dependent horizon design alleviates the imbalance caused by differences in the attitude channels’ sensitivities to horizon length and improves estimation quality across the full dataset.

Regarding computational cost and memory footprint, it should be emphasized that our efficiency discussion is primarily based on comparisons between the proposed QR-based solver and its corresponding non-QR implementation under the ASS-UFIR formulation, rather than against traditional baseline filters with fundamentally different update structures. As shown in Table 3, replacing the baseline batch solver in ASS-UFIR with the Givens-rotation QR solver reduces the runtime from 471.420s to 381.794s, i.e., an improvement of approximately 19%, while preserving identical estimation accuracy (including the RA metric). Moreover, the QR factorization avoids explicitly forming and inverting the normal matrix and enables an in-place triangular update, thereby reducing the peak storage requirement when solving the overdetermined batch system. In addition, by restructuring the estimation system via state separation, the maximum batch system dimension is reduced by up to 44% relative to the unreduced formulation, further lowering memory demand. These benefits, together with initializing the horizon using $N_{\mathrm{init}}=5$ and $N_{\min}$ to shorten the initial warm-up period (instead of starting directly from $N=N_{\max}$), support the practical feasibility of deploying the proposed method on resource-limited platforms.

## 6. Conclusions

This paper proposed an adaptive-state-separated UFIR attitude estimation method for MARG sensors to address the practical limitation that a single UFIR horizon length *N* can be suboptimal when different attitude angles exhibit different (and sometimes conflicting) sensitivity to the horizon. In particular, roll and pitch are primarily constrained by the gravity direction measured by the accelerometer, whereas yaw relies more strongly on geomagnetic heading from the magnetometer, motivating-state-dependent horizon allocation. Using a quaternion-based formulation, we group the attitude-related states and assign distinct horizon windows; Euler-angle quantities are used only to guide the grouping/adaptation while maintaining a consistent quaternion representation. To accommodate time-varying vehicle dynamics, an angle-guided mechanism is further introduced for online horizon updating. Simulation and field experiments show that the proposed method achieves improved overall attitude accuracy relative to representative baselines across the full dataset, as reflected in both channel-wise RMSEs and the global RA metric. Moreover, within the ASS-UFIR formulation, replacing the baseline batch solver with a Givens-rotation QR solver reduces runtime without altering estimation accuracy, and the reduced-batch formulation reduces the size of the stacked overdetermined system to be solved, facilitating efficient implementation on resource-limited platforms. Future work will extend the approach to more complex models and operating conditions with stronger disturbances and broader motion regimes.

## Figures and Tables

**Table 1:** Comparison of RMSEs and computation time in constant-velocity simulations.

Algorithm	Roll [°]	Pitch [°]	Yaw [°]	RA [°]	Time [s]
SS-UFIR	0.171	0.174	0.448	0.504	0.48
UFIR-29	0.396	0.376	0.445	0.642	0.59
UFIR-5	0.173	0.178	0.726	0.766	0.07

**Table 2:** Comparison of RMSEs and computation time using simulated vehicle dynamics.

Algorithm	Roll [°]	Pitch [°]	Yaw [°]	RA [°]	Time [s]
ASSQR-UFIR	0.201	0.190	3.152	3.161	0.42
ASS-UFIR	0.201	0.190	3.152	3.161	0.56
SS-UFIR	0.201	0.190	3.152	3.161	0.55
UFIR-35	0.330	0.333	3.165	3.198	0.58
UFIR-5	0.201	0.190	3.187	3.196	0.07

**Table 3:** The RMSEs and time of attitude angles comparison in the experiment.

Algorithm	Roll [°]	Pitch [°]	Yaw [°]	RA [°]	Time [s]
ASSQR-UFIR	3.378	3.510	4.909	6.923	381.794
ASS-UFIR	3.378	3.510	4.909	6.923	471.420
UFIR-70	3.358	3.521	5.283	7.180	450.064
UFIR-5	3.674	3.721	4.867	7.150	20.586
AQUA-KF	3.889	3.975	7.661	13.946	10.086
Madgwick’s GDA	4.225	2.747	8.938	10.154	3.072
Mahony’s SO(3)	6.592	3.857	9.373	11.886	1.724

## Data Availability

https://github.com/LZP1027/Adaptive-state-seperated-UFIR.git.
